# Interventions for reducing caregiver burden in chronic dyspnea: a meta-analysis

**DOI:** 10.3389/fpubh.2025.1659063

**Published:** 2025-10-17

**Authors:** Fang Liu, Han Zhang, Shihan Li, Xianshan Wang, Xiaoqing Long, Yang Liu

**Affiliations:** Day Surgery Center of General Practice Medical Center, West China Hosptial of Sichuan Universtiy, Chengdu, China

**Keywords:** caregiver burden, chronic respiratory diseases, heart failure, COPD, asthma, interventions, meta-analysis

## Abstract

**Background:**

Caregivers of patients with chronic respiratory diseases, such as heart failure, chronic obstructive pulmonary disease, and asthma, often experience significant physical, emotional, and psychological strain. This study aims to evaluate the effectiveness of various interventions designed to reduce caregiver burden and improve caregiver well-being.

**Design:**

Meta-analysis of randomized controlled trials (RCTs).

**Methods:**

A comprehensive literature search was conducted across multiple electronic databases, identifying randomized controlled trials (RCTs) that assessed interventions aimed at reducing caregiver burden in caregivers of chronic dyspnea patients. A total of 25 RCTs, involving 2,425 participants, were included. The included studies evaluated a variety of interventions, including psychological support, education programs, and physical activity. Data were extracted and analyzed using standardized mean differences (SMD) to assess intervention effects, with heterogeneity and publication bias considered.

**Results:**

A total of 25 RCTs involving 2,425 participants were included in the meta-analysis. Interventions significantly reduced caregiver burden (SMD = −0.65, 95% CI −0.96 to −0.34) with notable heterogeneity (I^2^ = 82.7%). Subgroup analysis showed a more pronounced reduction in studies conducted in Asia (SMD = −0.80). Improvements were also observed across caregiver burden categories, with the most significant reduction in social burden (SMD = −1.07). Family function improved (SMD = 0.53), but no significant change in social support (SMD = 0.55) or quality of life (SMD = 0.16) was found. Anxiety (SMD = −0.28) showed no significant reduction. Stress (SMD = −0.59) and depression (SMD = −0.45) were significantly reduced. Sensitivity analysis confirmed the robustness of the results.

**Conclusion:**

Interventions significantly reduce caregiver burden, particularly in emotional, physical, and social aspects, with improvements in family function, stress, and depression. However, no substantial changes were observed in anxiety or quality of life. The evidence quality is moderate, and future studies should focus on improving methodological rigor and exploring long-term effects.

**Tweetable abstract:**

Caregivers of chronic respiratory disease patients face significant strain. Our meta-analysis of 25 RCTs (2,425 participants) found that interventions significantly reduce caregiver burden, especially in emotional, physical, and social aspects, improving family function, stress, and depression. However, anxiety and quality of life showed no substantial changes. Future research should focus on long-term effects and methodological rigor.

**Systematic review registration:**

https://www.crd.york.ac.uk/PROSPERO/, identifier CRD420251034352.

## Introduction

Chronic respiratory diseases, such as chronic obstructive pulmonary disease (COPD), heart failure, and asthma, are leading causes of morbidity and mortality worldwide (1). Globally, COPD, heart failure and asthma affect more than 550 million adults, accounting for over 7% of the world’s population and contributing to more than 6 million deaths annually, ranking among the top five causes of mortality (2). Among which, most of patients with moderate-to-severe disease rely on informal family caregivers, whose depression, physical comorbidities and lost labor further amplify the economic impact on health systems. Current international standards—namely the 2024 Global Initiative for Chronic Obstructive Lung Disease report (3), the 2023 European Society of Cardiology guidelines for HF (4), and the Global Initiative for Asthma strategy (5)—all recommend integrating patient self-management with caregiver support. Yet, these documents focus primarily on pharmacological and device-based management of patients, offering limited guidance on interventions specifically designed to alleviate caregiver burden. Across the continuum of COPD, heart failure and asthma care, family caregivers perform multifaceted tasks that extend from acute exacerbation management to long-term stability maintenance. Caregivers of patients with chronic dyspnea often experience high levels of physical, emotional, and psychological stress, which can lead to caregiver burnout and negatively impact their own health (6–9). The “caregiver-as-second-patient” framework advanced by Schulz and Sherwood posits that family caregivers of chronically ill patients constitute a distinct population at risk for parallel trajectories of physical morbidity, emotional distress, and diminished quality of life (10). Within this paradigm, caregivers are not merely ancillary resources for the patient, but rather “hidden patients” who require systematic screening, risk stratification, and evidence-based interventions in their own right. Consequently, any therapeutic strategy targeting patients with chronic breathlessness that overlooks the caregiver’s burden risks sub-optimal overall effectiveness. Given the growing global prevalence of chronic respiratory diseases, addressing caregiver burden has become an essential component of healthcare management.

Caregiver burden refers to the emotional, physical, and financial strain experienced by individuals who provide care for patients with chronic conditions (11, 12). In the context of chronic dyspnea, caregiver burden can be particularly pronounced due to the ongoing nature of the disease, the complexity of care, and the emotional toll of managing a patient’s condition over extended periods (6). High caregiver burden is associated with increased rates of anxiety, depression, stress, and decreased quality of life, which can further complicate caregiving and reduce the caregiver’s ability to provide optimal care (13). Therefore, identifying effective interventions that alleviate caregiver burden is crucial for improving both caregiver well-being and the overall caregiving process.

Numerous interventions have been proposed to reduce caregiver burden, ranging from psychological and emotional support to practical assistance and training in caregiving techniques (14, 15). However, the effectiveness of these interventions varies, and there is a need for a comprehensive understanding of which interventions are most effective in different contexts. This meta-analysis aims to assess the impact of various interventions on caregiver burden for individuals caring for patients with chronic dyspnea, by synthesizing data from randomized controlled trials (RCTs). Specifically, we focus on examining the effects of these interventions on emotional, social, financial, and physical dimensions of caregiver burden, as well as their impact on anxiety, stress, confidence, depression, family function, and quality of life.

By consolidating evidence from multiple studies, this meta-analysis seeks to provide a clearer understanding of the effectiveness of interventions in alleviating caregiver burden and improving the well-being of caregivers. The findings from this analysis may help inform healthcare policies and intervention strategies aimed at supporting caregivers, thereby enhancing the overall care and quality of life for both patients with chronic respiratory conditions and their caregivers.

## Methods

### Study design and overall framework

This study is a systematic review and meta-analysis designed to evaluate the efficacy of interventions in reducing caregiver burden among informal carers of patients with COPD, heart failure or asthma. The review was conducted in strict accordance with the Cochrane Handbook for Systematic Reviews of Interventions (version 6.4) and is reported following the PRISMA 2020 statement. The study protocol was prospectively registered with PROSPERO (registration number: CRD420251034352).

### Data sources and search strategy

A comprehensive literature search was conducted from inception to 18 April 2025 in multiple electronic databases, including PubMed, Cochrane Library, Embase, and Web of Science. The search strategy was designed to capture RCTs that evaluated the effects of interventions on caregiver burden for caregivers of patients with chronic dyspnea. The search terms included combinations of the following keywords: “caregiver burden,” “chronic dyspnea,” “chronic respiratory disease,” “heart failure,” “COPD,” “asthma,” “caregiver interventions,” and “randomized controlled trial.” Detailed strategy was demonstrated in Supplementary Table 1. Additionally, relevant gray literature, such as conference abstracts and dissertations, were reviewed to minimize publication bias. The reference lists of included studies and relevant review articles were also manually searched for additional studies that met the inclusion criteria.

### Inclusion and exclusion criteria

The inclusion criteria for this meta-analysis were as follows: (1) Population: Informal, unpaid adult caregivers (older than 18 years) of community-dwelling patients with chronic respiratory diseases, including heart failure, COPD, asthma, or other chronic dyspnea conditions. (2) Any type of intervention aimed at reducing caregiver burden, including psychological support, education programs, training in caregiving techniques, or pharmacological treatments. (3) Studies were required to report at least one of the following outcomes: caregiver burden, anxiety, stress, depression, family function, quality of life, social support, or confidence in managing stress. (4) Studies had to be RCTs. (5) The research was published from inception to 18 April 2025. Exclusion criteria were applied to remove non-randomized trials, observational studies, and studies that did not involve structured interventions or report on caregiver burden or related outcomes. Additionally, studies involving caregivers of patients with diseases unrelated to chronic dyspnea or respiratory diseases were excluded. Finally, the entire selection process was managed in EndNote X9.

### Quality assessment and data extraction

Two reviewers independently assessed the quality of the included studies using the RoB 2.0 tool for randomized trials, which evaluates risk of bias across five domains: randomization process, deviations from intended interventions, missing outcome data, measurement of outcomes, and selection of the reported result. During the assessment, the randomization process was assessed by random allocation and baseline differences, the deviation from intended interventions was assessed by application of blinding. Any disagreements between the reviewers were resolved through discussion or consultation with a third reviewer. Data extraction was performed independently by two reviewers using a pre-designed data extraction form. The following data were extracted from each included study: study characteristics, population characteristics, intervention details, and outcomes.

### Statistical analysis

All statistical analyses were conducted using Stata 15 software. The effect size for continuous outcomes was expressed as standardized mean difference (SMD). A negative SMD indicates a reduction in caregiver burden or improvement in outcomes in the experimental group compared to the control group, while a positive SMD indicates the opposite. Heterogeneity among studies was assessed using the I^2^ statistic and Cochran’s Q test. If significant heterogeneity was found (I^2^ > 50%), a random-effects model was used to pool the effect sizes; otherwise, a fixed-effects model was used. Subgroup analyses were conducted to explore potential sources of heterogeneity based on factors such as study region, type of chronic respiratory disease, and duration of the intervention. Sensitivity analyses were performed by sequentially removing each study from the analysis to assess the robustness of the findings. Publication bias was assessed using Egger’s test when more than 10 studies contributed to a meta-analysis, and the trim-and-fill method was applied if publication bias was detected. The overall certainty of the evidence was assessed using the Grading of Recommendations Assessment, Development, and Evaluation (GRADE) system. Finally, Test Sequential Analysis (TSA) was employed to determine the required information size (RIS) and visualize the cumulative Z-curve to ensure statistical significance and rule out random variation in the results. The significance level for all statistical tests was set at *p* < 0.05.

### Ethical considerations

This meta-analysis was based on previously published studies, so ethical approval was not required. However, all studies included in the analysis were required to have received appropriate ethical approval by their respective institutional review boards or ethics committees.

## Results

### Research selection

**Figure 1:**
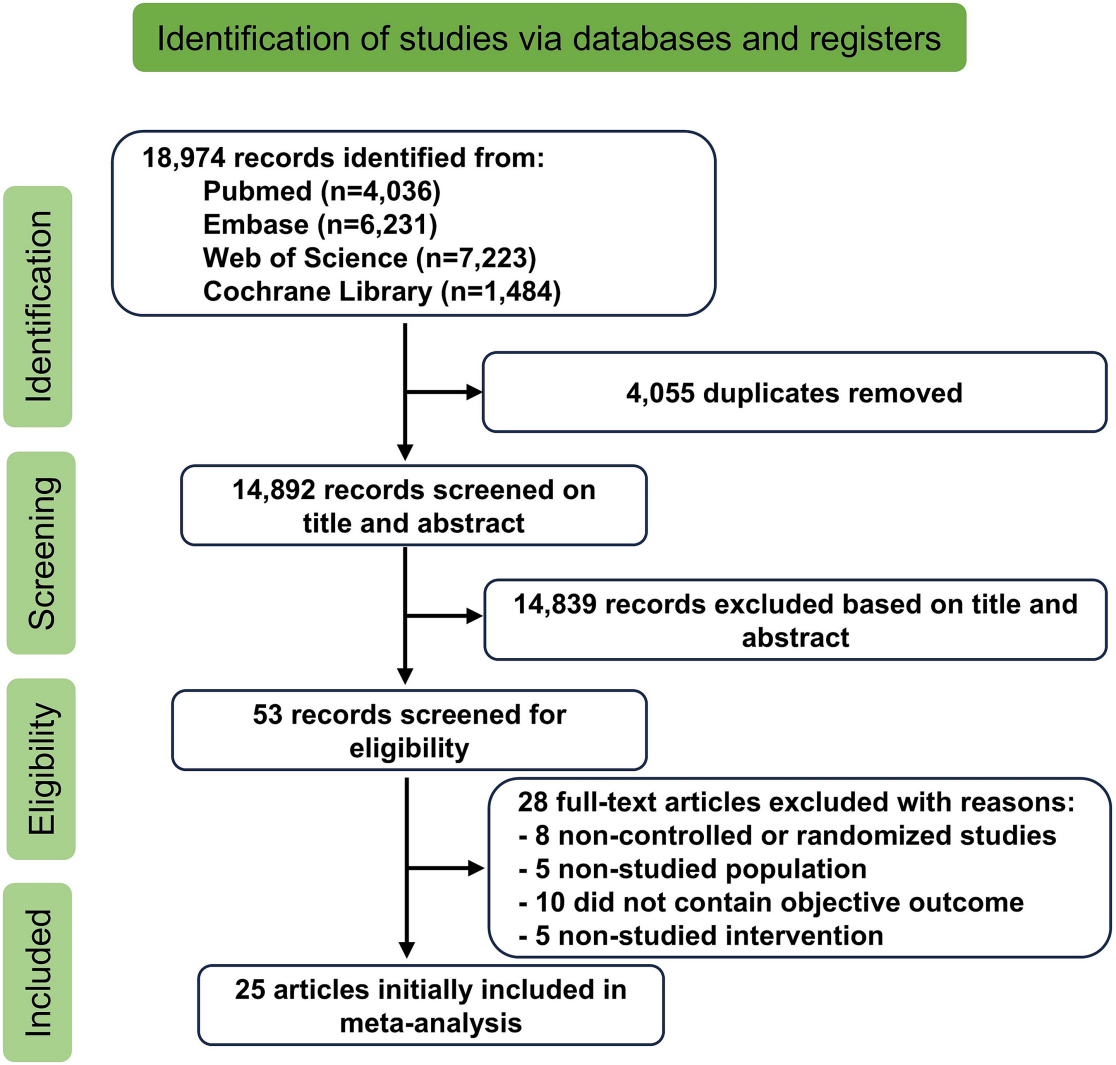
Flowchart illustrating the systematic review process.

A total of 18,974 studies were initially identified through database searches and other sources. After removing duplicates, 14,892 studies remained. Following a screening of titles and abstracts, 14,839 unrelated studies were excluded. A full-text review resulted in the exclusion of an additional 28 studies for various reasons: 8 were not RCTs (16–23), 5 involved unrelated populations (24–28), 10 did not measure the intended outcomes (29–38), and 5 lacked the relevant interventions (39–43). In total, 25 RCTs were included in the systematic evaluation (44–68). The study selection process is illustrated in Figure 1.

### Description of included studies

**Figure 2:**
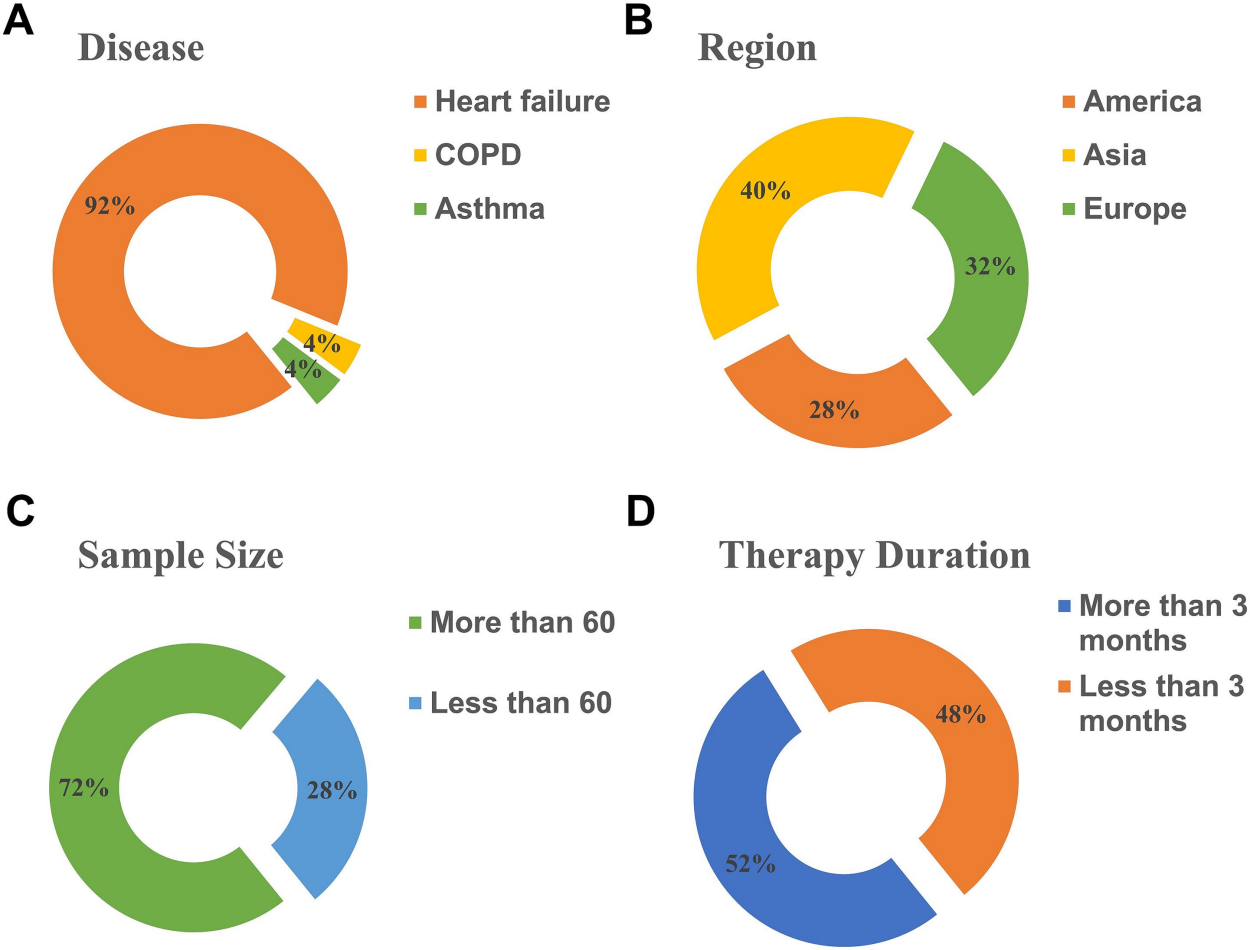
Characteristics of studies included in the analysis. **(A)** Diseases. **(B)** Region. **(C)** Sample size. **(D)** Therapy duration.

Twenty-five studies were included, involving 2,425 participants. Of these, 23 studies focused on caregivers of patients with heart failure, 1 study focused on COPD, and 1 study on asthma. Seven studies were conducted in the United States, 10 in Asia, and 8 in Europe. Sample sizes ranged from 20 to 510 participants; 18 studies included more than 60 individuals, while 7 studies had fewer than 60. Regarding the duration of interventions, 12 studies lasted less than 3 months, while 13 lasted longer than 3 months. The basic characteristics of the included studies are summarized in Figure 2 and Table 1.

**Table 1:** Characteristics of included studies.

Author	Year	Disease	Region	Age in intervention group	Age in control group	Gender (male/female)	Sample	Intervention	Control	Duration (weeks)	Follow-up time (weeks)	Frequency
Arash Marzban	2024	Heart failure	Iran	42.02 ± 12.21	43.06 ± 11.54	Intervention: 13/33 Control: 17/28	91	Emotional freedom techniques, EFT	Standard Care	4	4	Twice a week
Atefeh Alae	2024	Heart failure	Iran	41.56 ± 10.62	40.49 ± 10.80	Intervention: 9/36 Control: 19/26	90	COPE model education	Standard Care	4	12	Four times
Barbara Riegel	2023	Heart failure	USA	55.4 ± 13.78	55.3 ± 13.55	Intervention: 19/106 Control: 18/107	250	Virtual health coaching intervention	Standard Care	24	24	Ten times
Boyoung Hwang	2022	Heart failure	South Korea	55.62 ± 13.54	54.46 ± 17.61	Intervention: 5/10 Control: 9/4	30	Cognitive behavioral therapy, CBT	Standard Care	8	8	Once a week
*Canan Demir Barutcu*	2016	Heart failure	Turkey	52.38 ± 12.67	57.00 ± 10.71	Intervention: 11/23 Control: 7/28	69	Caregivers of individuals	Standard Care	12	24	Once a week
Chim C Lang	2017	Heart failure	UK	71.8(9.9)	76.0(6.6)	Intervention: 9/16 Control: 14/11	50	REACH-HF	Standard Care	12	24	Twice a week
Döndü ¸Sanlıtürk	2023	Asthma	Turkey	18–40:15(50%) 41–64:15(50%)	18–40:15(50%) 41–64:15(50%)	Intervention: 10/20 Control: 10/20	60	Home visit program	Standard Care	12	12	Five times in three months
Gerard J. Molloy	2005	Heart failure	UK	65.0 ± 15	61.6 ± 14	Intervention: 8/24 Control: 13/17	62	Effects of an exercise intervention	Standard Care	12	12	Three times in 12 weeks
Giulia Locatelli	2023	Heart failure	Italy	57(44–68)	53(42–64)	Intervention: 42/135 Control: 45/133	510	Motivational interviewing	Standard Care	8	48	Four times
Katherine Doyon	2024	Heart failure	USA	55.2 ± 16	59.9 ± 15	Intervention: 10/14 Control: 8/36	101	CASA intervention	Standard Care	48	48	Three times a year
Li-Chi Chiang	2012	Heart failure	China	18–39:6(20%) 40–59:9(30%) 60–79:14(46.7%) > =80:1(3.3%)	18–39:4(13.3%) 40–59:17(56.7%) 60–79:7(23.3%) > =80:2(6.7%)	Intervention: 7/23 Control: 10/20	60	Participate in either telehealth care	Standard Care	4	4	Once a day
Linda Clements	2020	Heart failure	USA	<=50:2(22%) 50 < =60:3(32%) 60 < =70:11(69%) > 70:3(16%)	<=50:7(38%) 50 < =60:4(22%) 60 < =70:5(31%) > 70:2(11%)	Intervention: 12/7 Control: 14/4	37	Heart failure education	Standard Care	4	4	Three times
Loghman Khaninezhad	2023	Heart failure	Iran	5.25 ± 34.27	4.86 ± 34.09	Intervention: 20/25 Control: 18/27	90	Pender’s Health Promotion Model care	Standard Care	7	7	Twice a week
Maria Liljeroos	2016	Heart failure	Switzerland	67.1 ± 12.1	69.5 ± 10.5	Intervention: 22/49 Control: 16/68	155	A three session nurse-led psycho-educational program	Standard Care	12	96	Once a week
Maria Thodi	2023	Heart failure	Greece	61.3 ± 14.8	59.6 ± 13.8	Intervention: 7/23 Control: 2/25	57	Combination of home visits and telephone sessions	Standard Care	24	24	Once a week
Martha Abshire Saylor	2023	Heart failure	USA	55.8 ± 19.6	62.1 ± 13.9	Intervention: 1/11 Control: 1/11	24	Caregiver Support	Standard Care	10	16	Five times
Mohaddeseh Namjoo MSc	2021	Heart failure	Iran	20–40:6(12%) 41–55:16(32%) 56–70:19(38%) 71–85:9(18%)	20–40:3(6%) 41–55:25(50%) 56–70:18(36%) 71–85:4(8%)	Intervention: 25/25 Control: 26/24	100	Tele nursing Intervention	Standard Care	4	4	Twice a week
Rebecca Gary	2018	Heart failure	USA	54 ± 10	57 ± 14	Intervention: 2/8 Control: 6/42	127	Psychoeducation plus exercise	Standard Care	24	24	Three times a week
Seyyed Abolfazl Vagharseyyedin	2022	COPD	Iran	38.74 ± 13.73	42.16 ± 15.71	Intervention: 20/23 Control: 15/29	92	Caregiver Educational Program	Standard Care	1	8	Four times
Shahram	2014	Heart failure	Iran	20–39:21(50%) 40–59:20(47.7%) > 60:1(2.4%)	20–39:21(46.7%) 40–59:24(53.3%) > 60:0(0)	Intervention: 10/32 Control: 7/38	87	Education and family support	Standard Care	4	12	Weekly
Susanna Ågren	2015	Heart failure	Sweden	67(7)	66(8)	Intervention: 4/21 Control: 1/16	42	Psycho-educational intervention	Standard Care	24	12	Three times
Ubolrat Piamjariyakul	2015	Heart failure	USA	60.8(14.5)	63.7(13.1)	Intervention: 2/8 Control: 1/9	20	FamHFcare	Standard Care	24	24	Once a week
Ubolrat Piamjariyakul	2024	Heart failure	American	65.57(13.38) (40–85)	65.77(14.51) (32–88)	Intervention: 5/16 Control: 4/14	39	FamPALcare intervention	Standard Care	12	24	Five times
Weiling Yang	2023	Heart failure	China	58.78 ± 10.82	55.47 ± 13.59	Intervention: 10/22 Control: 16/16	64	Caregiver-mediated online dignity therapy	Standard Care	4	8	Three times a week
Xiaolin Hu	2016	Heart failure	China	<=40:28 41–49:20 50–59:4 > =60:7	<=40:21 41–49:25 50–59:10 > =60:3	Intervention: 24/35 Control: 26/33	118	Multidisciplinary supportive program	Standard Care	12	12	Once a week

### Risk of bias assessment

**Figure 3:**
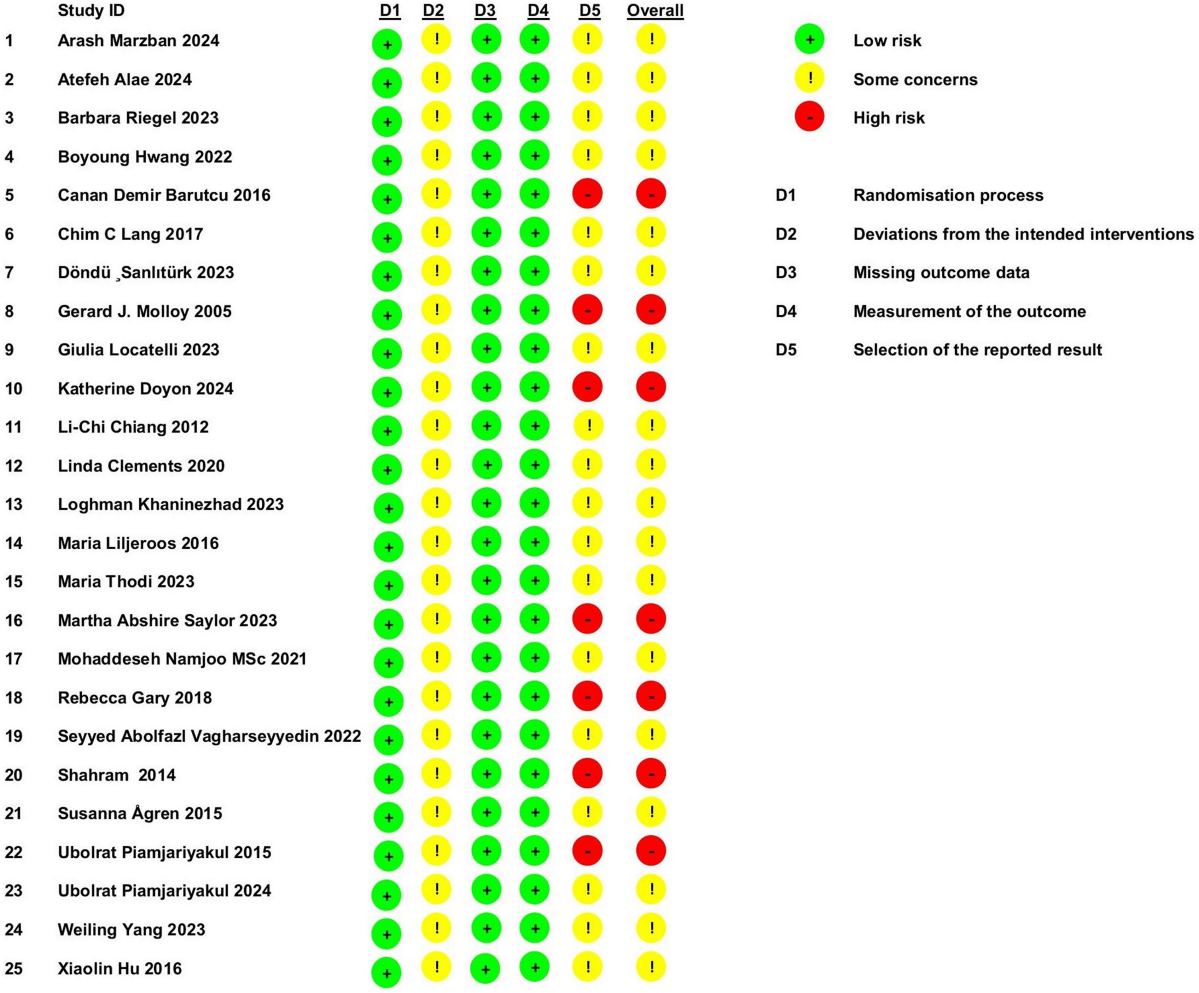
Assessment of bias risk and quality in the selected studies.

According to the RoB 2.0 tool for assessing the risk of bias in randomized trials, the following observations were made: All studies employed random allocation and showed no baseline differences, suggesting a low risk of bias in the randomization process. However, none of the studies employed blinding, raising concerns about potential deviations in the delivery of the interventions. All studies reported complete data, minimizing the risk of bias in this domain. Objective outcome measures were used, which further reduced the risk of bias in outcome assessment. Seven studies were deemed high risk due to inadequate methods for measuring outcomes, and 18 studies raised concerns about selective reporting, lacking adequate justification for outcome measures. These findings are summarized in Figure 3.

### Meta-analysis

#### Improvement in caregiver burden

**Figure 4:**
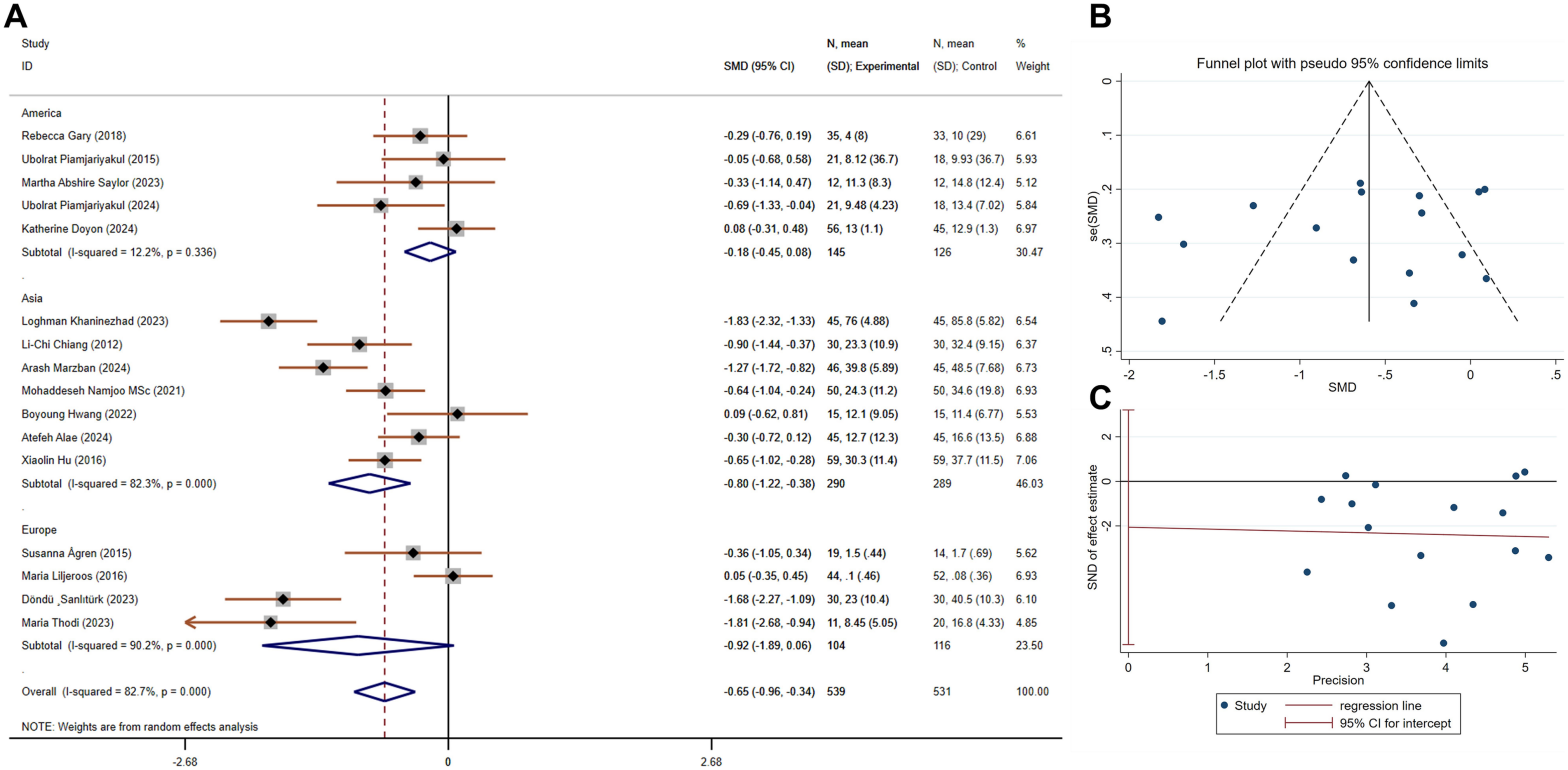
The effect size and 95% CI for intervention on caregiver burden. **(A)** Forest plot. **(B)** Funnel plot. **(C)** Egger’s test results. SMD, standard mean differences; CI, confidence intervals.

Caregiver burden is a critical measure for understanding the emotional, physical, and financial strain experienced by those caring for patients with chronic conditions like chronic dyspnea. A reduction in caregiver burden can prevent burnout, enhance caregivers’ ability to provide care, and potentially improve patient outcomes by allowing caregivers to continue their role with better health and support. A total of 16 studies, involving 1,070 participants (539 in the experimental group and 531 in the control group), compared caregiver burden. These studies showed significant heterogeneity (*p* < 0.001, I^2^ = 82.7%). Using a random-effects model, the pooled SMD was −0.65 (95% CI −0.96 to −0.34), indicating a statistically significant reduction in caregiver burden in the experimental group compared to the control group. A subgroup analysis based on study region revealed that studies conducted in Asia showed a more pronounced reduction in caregiver burden (SMD = −0.80, 95% CI −1.22 to −0.38), with some reduction in heterogeneity (Figure 4A). No publication bias was detected via Egger’s test (*p* = 0.416, Figures 4B,C).

#### Improvement in categories of caregiver burden

**Figure 5:**
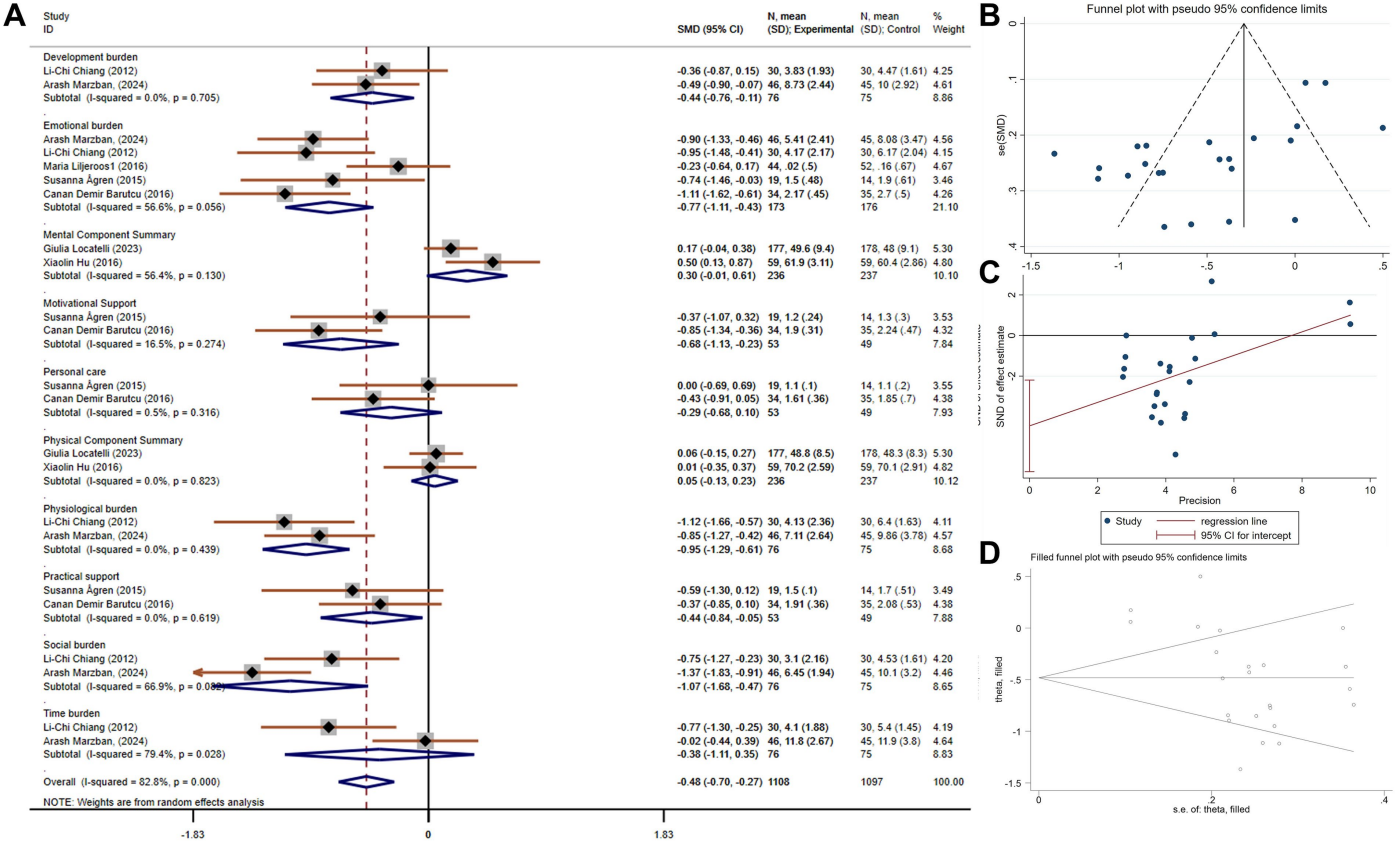
The effect size and 95% CI for intervention on categories of caregiver burden. **(A)** Forest plot. **(B)** Funnel plot. **(C)** Egger’s test results. **(D)** Trim and fill method application. SMD, standard mean differences; CI, confidence intervals.

Caregiver burden can be broken down into various categories, such as emotional, social, financial, and physical burden. Each of these categories impacts the caregiver in different ways. Twenty-three studies, involving 2,205 participants (1,108 in the experimental group and 1,097 in the control group), compared categories of caregiver burden. These studies exhibited substantial heterogeneity (*p* < 0.001, I^2^ = 82.8%). The pooled SMD, derived from a random-effects model, was −0.48 (95% CI −0.70 to −0.27), indicating significant improvement across all categories of caregiver burden in the experimental group. A subgroup analysis by category of burden showed the most significant improvement in social burden (SMD = −1.07, 95% CI −1.68 to −0.47, Figure 5A). Publication bias was present in this analysis (*p* = 0.001, Figures 5B,C), but further examination with the trim-and-fill method indicated that this bias did not substantially affect the results (i.e., no trimming was necessary as the data remained unchanged, Figure 5D).

#### Improvement in family function

**Figure 6:**
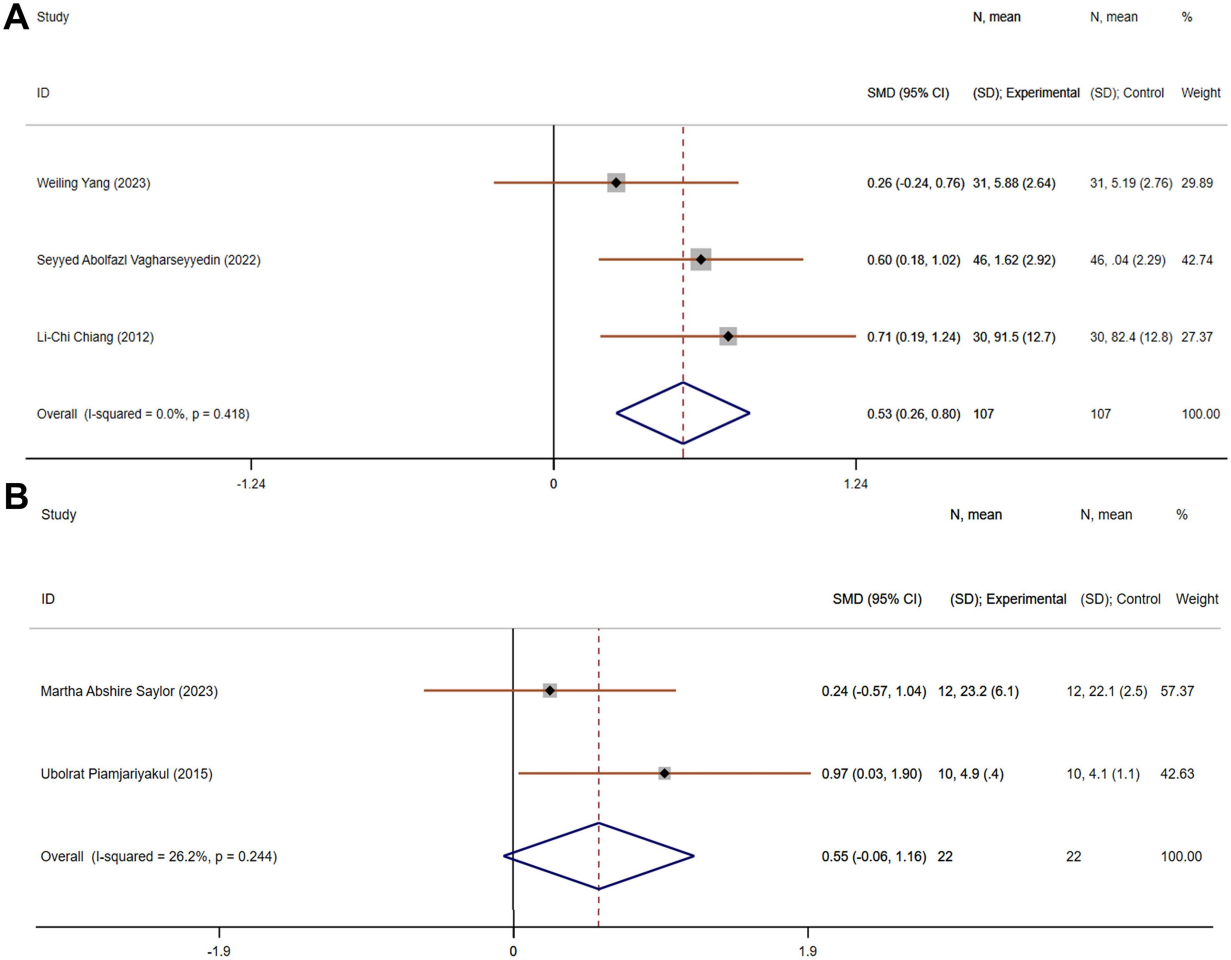
The effect size and 95% CI for intervention on family function and social support. **(A)** Forest plot of family function. **(B)** Forest plot of social support. SMD, standard mean differences; CI, confidence intervals.

Family function reflects the dynamics within a caregiver’s household and the ability of family members to support one another. Improving family function can strengthen the emotional and logistical support systems for caregivers, making caregiving tasks more manageable and reducing the overall strain. Four studies comparing family function included 214 participants (107 in each group). There was no heterogeneity (*p* = 0.418, I^2^ = 0). Using a fixed-effects model, the pooled SMD was 0.53 (95% CI 0.26 to 0.80), indicating a significant improvement in family function in the experimental group compared to the control group (Figure 6A).

#### Improvement in social support

Social support is crucial for caregivers, as it provides emotional reassurance, practical help, and an outlet for stress. Two studies, with a total of 44 participants (22 in each group), assessed social support. These studies exhibited acceptable heterogeneity (*p* = 0.244, I^2^ = 26.2%). The pooled SMD, derived from a fixed-effects model, was 0.55 (95% CI −0.06 to 1.16), suggesting no statistically significant improvement in social support in the experimental group compared to the control group (Figure 6B).

#### Improvement in anxiety

**Figure 7:**
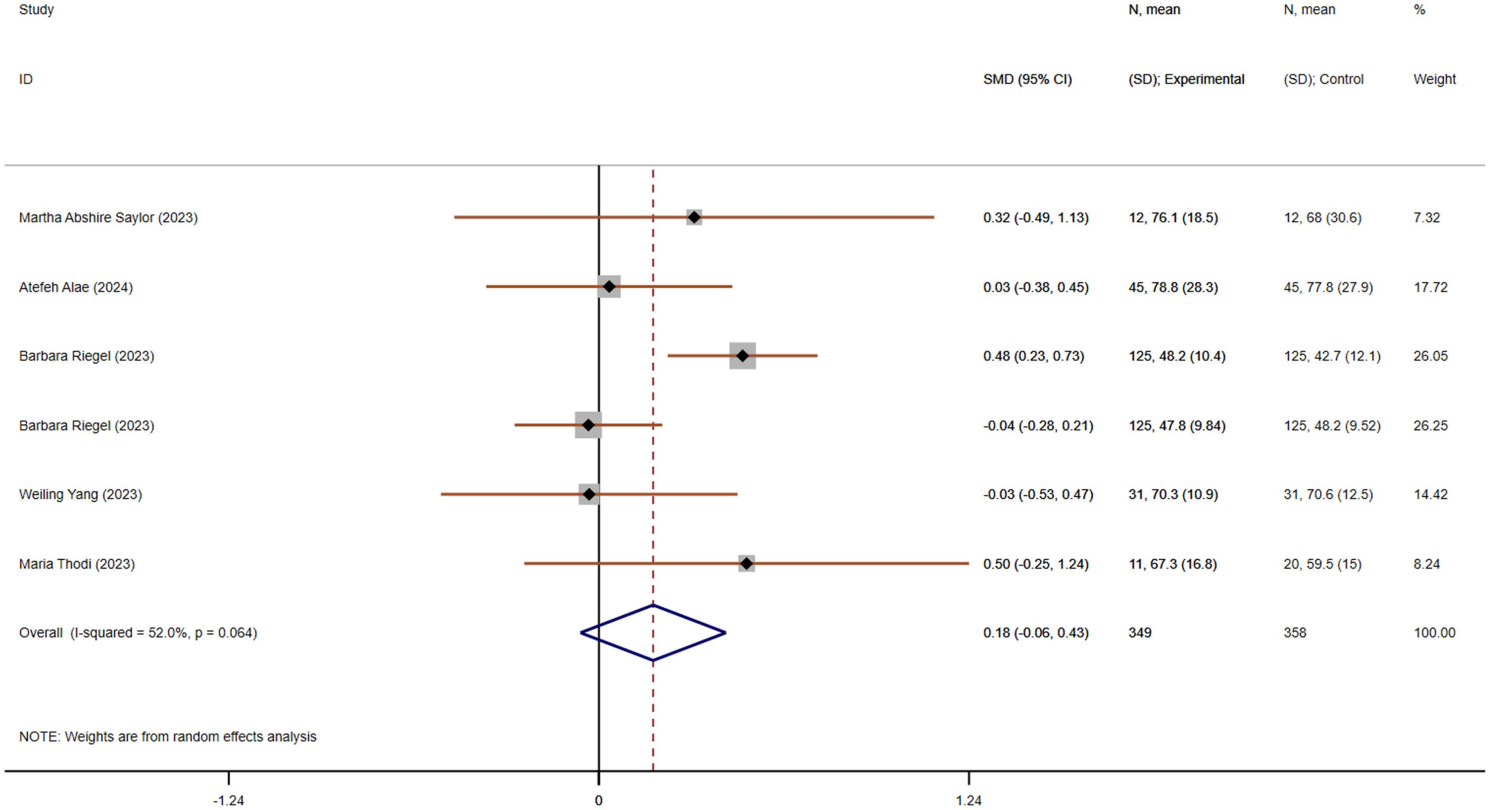
The effect size and 95% CI for intervention on anxiety. SMD, standard mean differences; CI, confidence intervals.

Anxiety is a common emotional response among caregivers, particularly those caring for individuals with chronic conditions. Chronic stress and anxiety can negatively affect caregivers’ physical and mental health, leading to fatigue, depression, and burnout. Seven studies, with a total of 682 participants (342 in each group), compared anxiety. These studies showed moderate heterogeneity (*p* = 0.003, I^2^ = 69.7%). The pooled SMD, derived from a random-effects model, was −0.28 (95% CI −0.60 to 0.05), indicating no statistically significant reduction in anxiety in the experimental group compared to the control group (Figure 7). Subgroup analysis based on the region or intervention duration showed a decreased heterogeneity, indicating region or intervention duration might be the cause of heterogeneity (Supplementary Figure 1).

#### Improvement in stress

**Figure 8:**
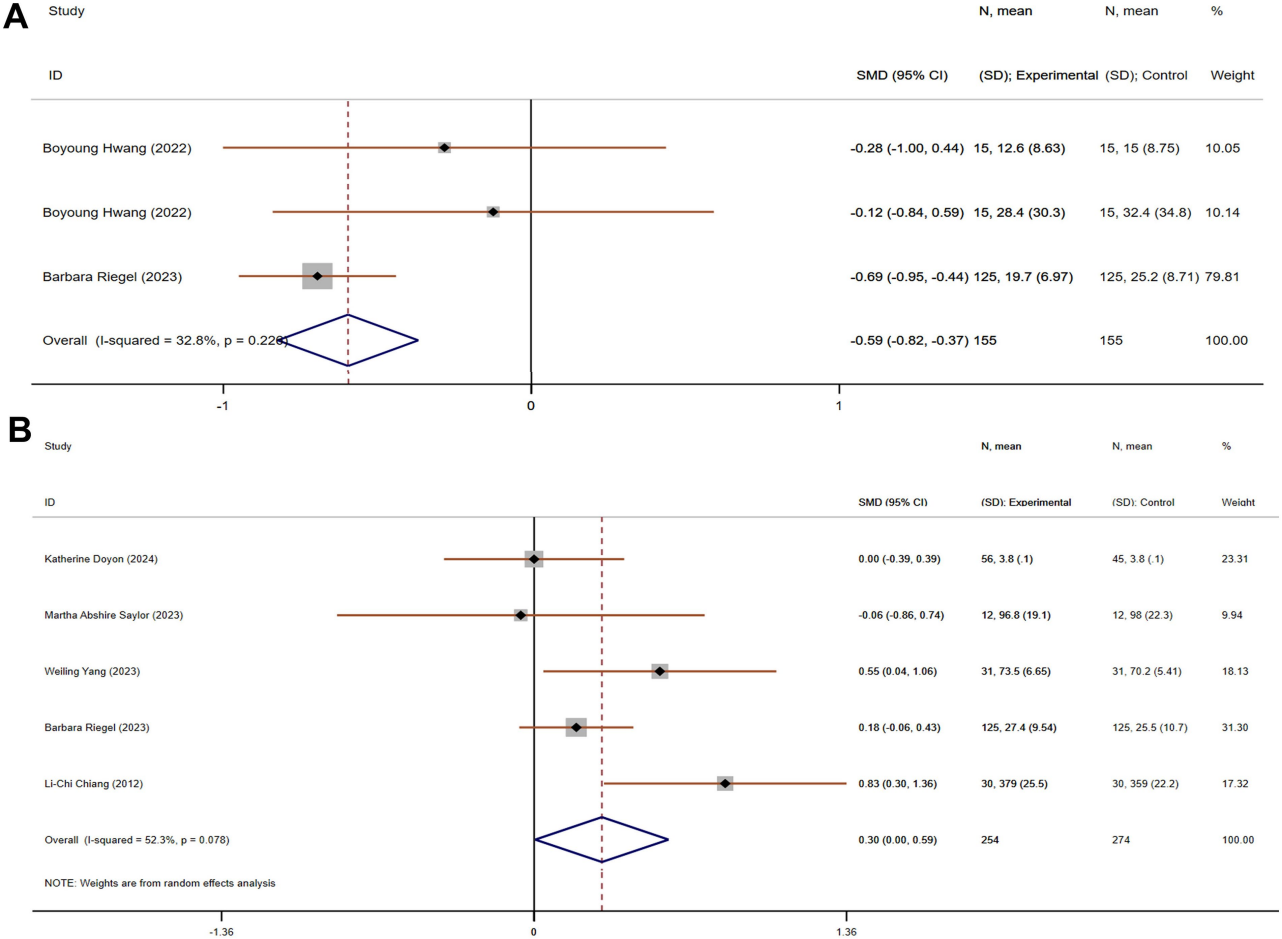
The effect size and 95% CI for intervention on stress and confidence facing stress. **(A)** Forest plot of family function. **(B)** Forest plot of social support. SMD, standard mean differences; CI, confidence intervals.

Stress is a key factor contributing to caregiver burden, particularly for those caring for individuals with chronic, life-limiting conditions like chronic dyspnea. Chronic stress can lead to various health issues, including cardiovascular problems, insomnia, and depression. Three studies, including 310 participants (155 in each group), compared stress. These studies showed acceptable heterogeneity (*p* = 0.228, I^2^ = 32.8%). The pooled SMD, derived from a fixed-effects model, was −0.59 (95% CI −0.82 to −0.37), indicating a statistically significant reduction in stress in the experimental group compared to the control group (Figure 8A).

#### Improvement in confidence for facing stress

Confidence in managing stress is an important predictor of how well caregivers can cope with their responsibilities. Caregivers with higher confidence are less likely to feel overwhelmed and are better equipped to handle challenging situations. Five studies, with 528 participants (254 in the experimental group and 274 in the control group), compared confidence for facing stress. These studies showed acceptable heterogeneity (*p* = 0.078, I^2^ = 52.3%). Using a random-effects model, the pooled SMD was 0.30 (95% CI 0.00 to 0.59), suggesting a statistically significant improvement in confidence for facing stress in the experimental group compared to the control group (Figure 8B).

#### Improvement in depression

**Figure 9:**
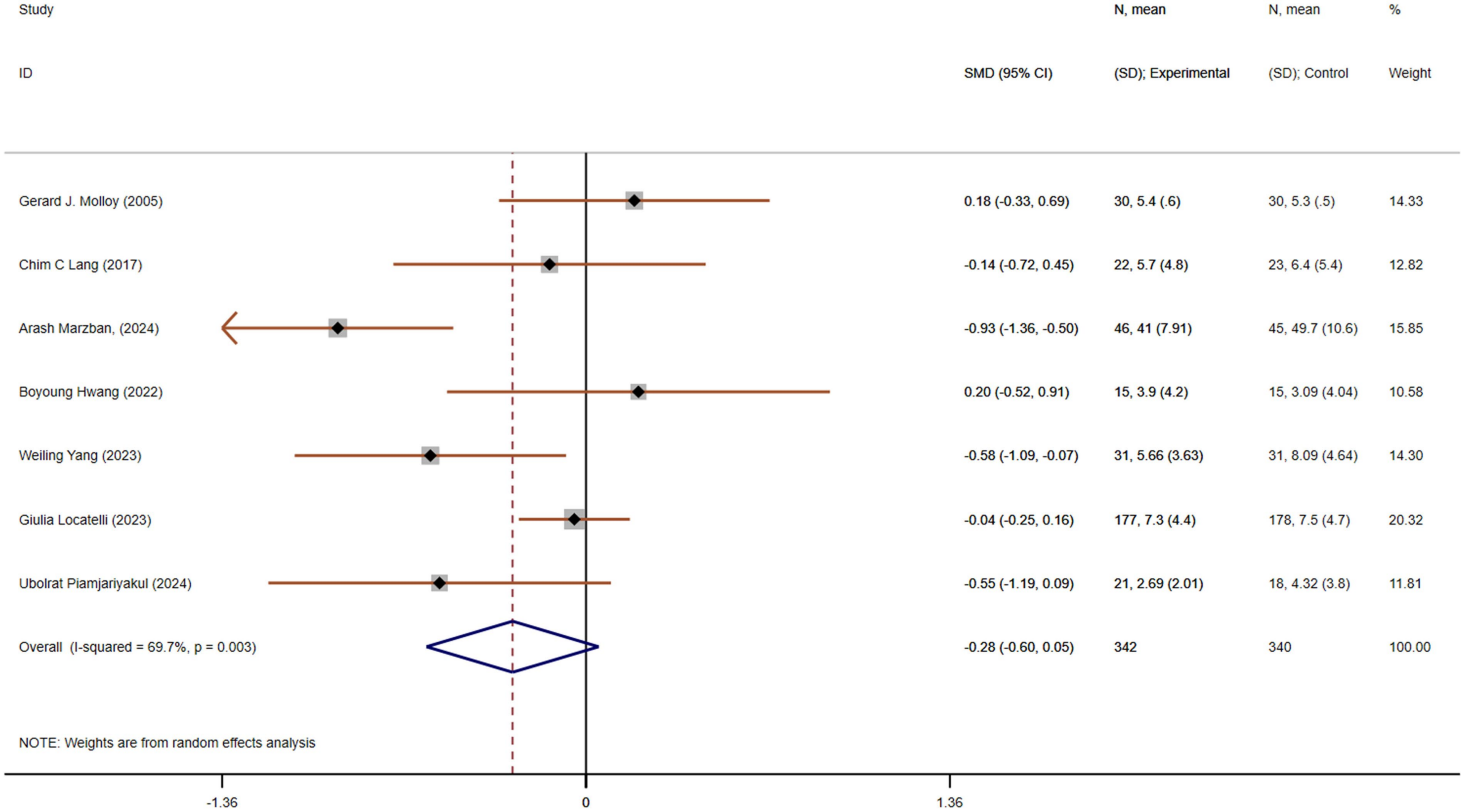
The effect size and 95% CI for intervention on depression. SMD, standard mean differences; CI, confidence intervals.

Depression is a significant concern for caregivers, as the emotional toll of caregiving can lead to or exacerbate existing mental health issues. Nine studies, involving 860 participants (434 in the experimental group and 426 in the control group), compared depression. These studies showed moderate heterogeneity (*p* < 0.001, I^2^ = 75.1%). The pooled SMD, derived from a random-effects model, was −0.45 (95% CI −0.76 to −0.14), indicating a statistically significant reduction in depression in the experimental group compared to the control group (Figure 9). Subgroup analysis based on the region or intervention duration showed a decreased heterogeneity, indicating region or intervention duration might be the cause of heterogeneity (Supplementary Figure 1).

#### Improvement in quality of life

**Figure 10:**
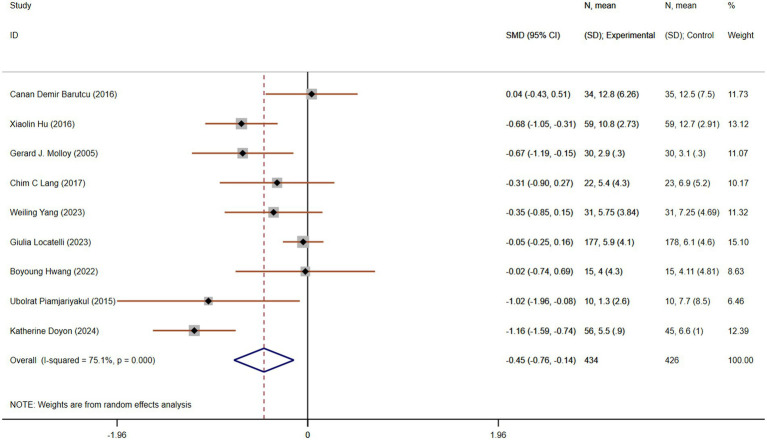
The effect size and 95% CI for intervention on quality of life. SMD, standard mean differences; CI, confidence intervals.

Quality of life is a comprehensive measure that captures the physical, emotional, and social well-being of caregivers. Their role over the long term. Six studies, with 707 participants (349 in the experimental group and 358 in the control group), compared quality of life. These studies showed acceptable heterogeneity (*p* = 0.064, I^2^ = 52.0%). The pooled SMD, derived from a random-effects model, was 0.16 (95% CI −0.06 to 0.43), suggesting no statistically significant improvement in quality of life in the experimental group compared to the control group (Figure 10). Subgroup analysis based on the region or intervention duration showed a decreased heterogeneity, indicating region or intervention duration might be the cause of heterogeneity (Supplementary Figure 1).

### Sensitivity analysis

**Figure 11:**
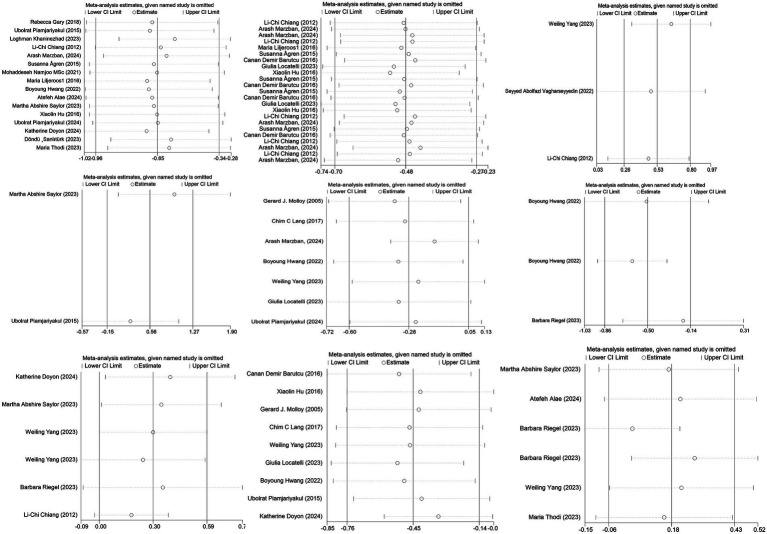
Sensitivity analysis of the study results. CI, confidence intervals.

Sensitivity analyses, performed by systematically excluding one study at a time, revealed consistent results, without significant changes in the outcomes (see Figure 11). This reinforces the validity and reliability of the analysis, indicating that the overall findings are robust and not unduly influenced by any single study.

### Certainty of the evidence

Based on the assessment of bias risk, reporting bias, and consistency across trials, the evidence was graded as follows: moderate quality for caregiver burden, family function, social support, anxiety, stress, confidence for facing stress, depression, and quality of life. The evidence for the categories of caregiver burden was graded as very low quality (see Table 2). These quality assessments highlight areas where further research is needed to strengthen the evidence base.

**Table 2:** GRADE evidence profile.

Quality assessment						Quality
No. of studies	Risk of bias	Inconsistency	Indirectness	Imprecision	Other considerations	
Caregiver burden						
16	Serious	No serious inconsistency	No serious indirectness	No serious imprecision	None	⊕ ⊕ ⊕○ Moderate
Category of caregiver burden						
23	Serious	Serious^a^	No serious indirectness	No serious imprecision	Reporting bias^b^	⊕○○○ Very low
Family functions						
4	Serious	No serious inconsistency	No serious indirectness	No serious imprecision	None	⊕ ⊕ ⊕○ Moderate
Social support						
2	Serious	No serious inconsistency	No serious indirectness	No serious imprecision	None	⊕ ⊕ ⊕○ Moderate
Anxiety						
7	Serious	No serious inconsistency	No serious indirectness	No serious imprecision	None	⊕ ⊕ ⊕○ Moderate
Stress						
3	Serious	No serious inconsistency	No serious indirectness	No serious imprecision	None	⊕ ⊕ ⊕○ Moderate
Confidence for facing stress						
5	Serious	No serious inconsistency	No serious indirectness	No serious imprecision	None	⊕ ⊕ ⊕○ Moderate
Depression						
9	Serious	No serious inconsistency	No serious indirectness	No serious imprecision	None	⊕ ⊕ ⊕○ Moderate
Quality of life						
6	Serious	No serious inconsistency	No serious indirectness	No serious imprecision	None	⊕ ⊕ ⊕○ Moderate

a There is controversy in different studies.

b Egger’s test *P* < 0.05.

### Test sequential analyses

**Figure 12:**
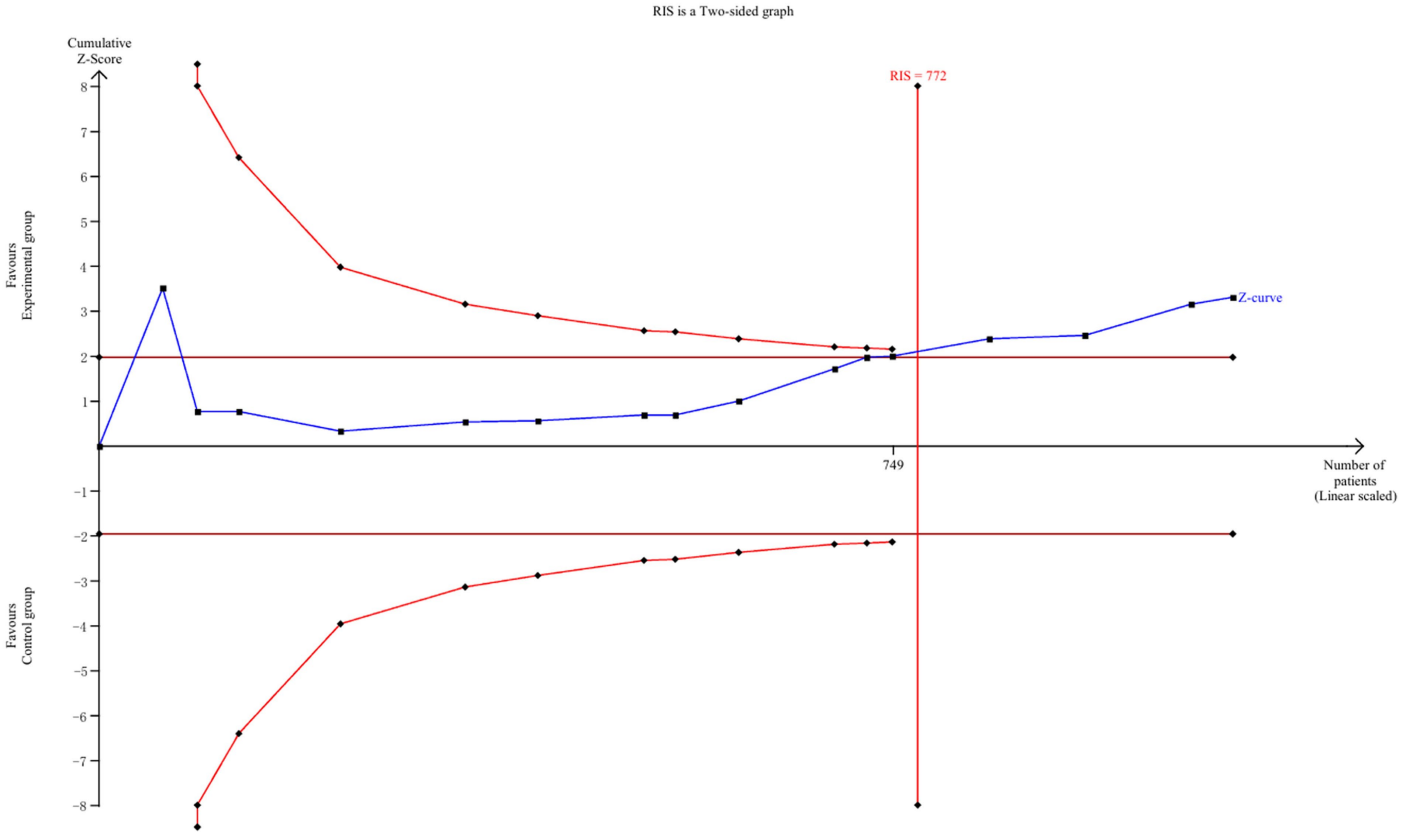
TSA for intervention on caregiver burden. TSA, trial sequential analysis; RIS, required information size.

The TSA boundary graph depicting the effects of interventions on caregiver burden for caregivers of chronic dyspnea patients, based on the RIS, is shown in the Figure 12. The RIS is 772, and the cumulative Z-curve crosses both the traditional boundary (Z = 1.96) and the RIS, suggesting that the experimental group experienced a lower burden than the control group, with false positive results ruled out.

## Discussion

This meta-analysis aimed to assess the impact of various interventions on the caregiver burden of individuals caring for patients with chronic dyspnea. A total of 25 RCTs were included, providing a comprehensive view of interventions aimed at alleviating the emotional, physical, and financial strain experienced by caregivers. The analysis identified significant improvements across multiple aspects of caregiver burden, with notable variation in the outcomes based on region, intervention type, and specific burden categories.

The pooled analysis of 16 studies involving 1,070 participants demonstrated a significant reduction in overall caregiver burden in the experimental group compared to the control group (SMD = −0.65). The substantial heterogeneity observed (I^2^ = 82.7%) suggests that various factors, such as study design, population characteristics, and types of interventions, may have contributed to the variability in results. Notably, studies conducted in Asia reported a more pronounced reduction in caregiver burden (SMD = −0.80), which may be attributed to cultural differences in caregiving practices, the type of interventions employed, or the specific characteristics of caregiver populations in these regions (69–71).

Further analysis of subcategories of caregiver burden revealed that the most significant improvements occurred in the social burden domain (SMD = −1.07), indicating that interventions were particularly effective in reducing the social strain caregivers experience. This is consistent with previous research that highlights the social isolation and lack of support often experienced by caregivers (72, 73). Interestingly, the reduction in emotional burden was less pronounced, suggesting that while caregivers may experience relief from practical or logistical aspects of caregiving, emotional support interventions may require more targeted approaches (74, 75). The categorization of caregiver burden allows for a nuanced understanding of the areas most impacted by interventions. It also highlights the need for multifaceted interventions that address not only the physical and financial aspects of caregiving but also the emotional and social dimensions. As caregivers are often under stress due to a lack of social support and emotional resources, interventions focusing on improving emotional well-being and social interactions may offer a more holistic approach to caregiver support (76, 77).

Improvements in family function were observed across four studies, with a significant positive effect (SMD = 0.53), suggesting that caregiver interventions can strengthen family dynamics and support systems. However, the effect on social support was less clear, with no statistically significant improvement (SMD = 0.55). These findings underscore the importance of targeting both individual caregivers and their broader support networks. While family function improved, social support interventions might require more intensive or sustained efforts to foster meaningful changes in caregivers’ social environments (78, 79).

The analysis also evaluated several psychological outcomes, including anxiety, stress, and depression. Statistically significant reductions in stress (SMD = −0.59) and depression (SMD = −0.45) were observed, which align with the broader literature suggesting that caregiving for patients with chronic conditions can exacerbate these mental health issues (77, 80). However, no significant reduction in anxiety was found (SMD = −0.28), which may reflect the complexity of anxiety as an emotional response and the challenge of addressing it through short-term interventions. It is possible that longer-term or more specific interventions are necessary to achieve meaningful reductions in caregiver anxiety (81). Confidence for managing stress was also improved (SMD = 0.30), which is an encouraging outcome. Increased confidence is a protective factor against caregiver burnout and may contribute to long-term well-being, enabling caregivers to handle stress more effectively (82, 83). This finding highlights the importance of equipping caregivers with the skills and strategies to manage the challenges they face.

No significant improvement in quality of life (SMD = 0.16) was found, suggesting that while interventions can alleviate caregiver burden in specific areas, they may not always lead to broad improvements in overall quality of life. It is possible that quality of life is influenced by factors beyond caregiver burden, including the severity of the patient’s condition, social and economic support, and personal coping strategies (84). Further research may explore how different types of interventions interact with these factors to affect caregivers’ overall quality of life.

### Limitation

This meta-analysis provides strong evidence for the positive impact of interventions on reducing caregiver burden in chronic dyspnea caregivers, particularly in terms of emotional, physical, and social strain. However, there are some limitations that could not be ignored. In the 25 included studies, only 8 studies from Europe, and 7 from the United State of America. This may limit the generalizability of our findings to European or North-American populations. This geographical imbalance partly reflects the growing research interest in caregiver burden across Asia. Given the differences in disease epidemiology, healthcare systems, and family-centered care cultures between regions, well-designed RCTs in Europe and the Americas are warranted to confirm the external validity of the observed effects. In addition, significant variability in study quality, intervention types, and outcome measures highlights the need for more rigorous and standardized trials in this field. Future research should focus on improving the methodological quality of RCTs, particularly in terms of blinding, outcome measurement, and reporting. Additionally, more studies are needed to explore the long-term effects of caregiver interventions, particularly on anxiety and quality of life, as these factors may require more sustained and targeted approaches.

## Conclusion

This meta-analysis provides compelling evidence that various interventions can significantly reduce caregiver burden in individuals caring for patients with chronic dyspnea, particularly in the areas of emotional, physical, and social strain. Significant improvements were observed in caregiver burden, family function, stress, depression, and confidence for managing stress, although no substantial changes were noted in anxiety or quality of life. The findings underscore the importance of tailored interventions that address specific dimensions of caregiver burden, especially social and emotional support, and aim to move the “caregiver-as-second-patient” concept from theory to routine practice and policy.

## Data Availability

The original contributions presented in the study are included in the article/Supplementary material, further inquiries can be directed to the corresponding author.
