# Supplementary material for: Interventions for reducing caregiver burden in chronic dyspnea: a meta-analysis
Source: Front Public Health. 2025 Oct 17;13:1659063. doi: 10.3389/fpubh.2025.1659063 (PMC12575251; doi:10.3389/fpubh.2025.1659063)
Supplement: Supplementary file 1 [file Supplementary_file_1.docx]

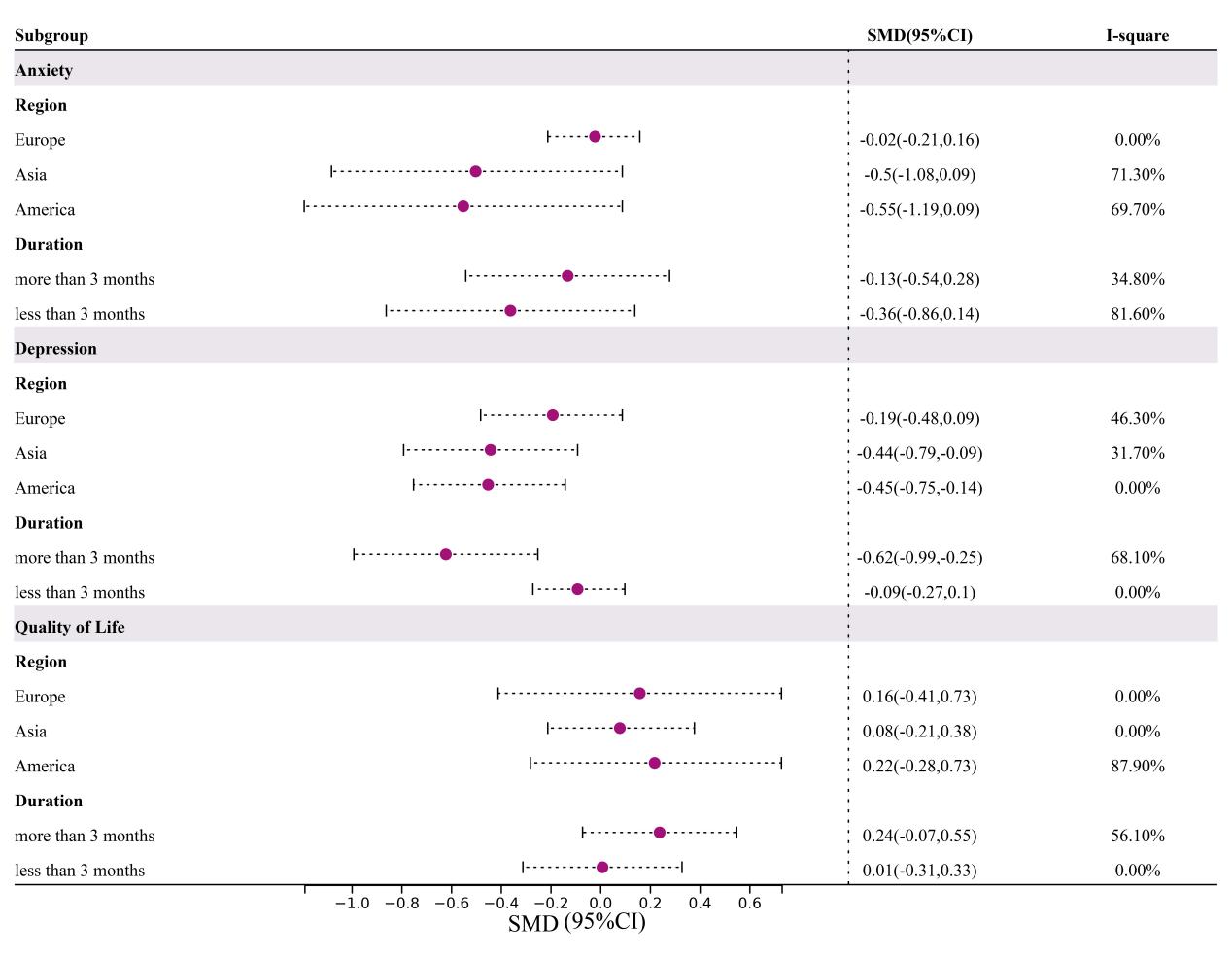
Supplementary Figure 1: Subgroup analysis according to region and intervention duration. SMD, standard mean deviation; CI, confidence interval.
