# Supplementary material for: Interventions for reducing caregiver burden in chronic dyspnea: a meta-analysis
Source: Front Public Health. 2025 Oct 17;13:1659063. doi: 10.3389/fpubh.2025.1659063 (PMC12575251; doi:10.3389/fpubh.2025.1659063)
Supplement: Supplementary file 2 [file Table_1.docx]

Supplementary Table 1: Searching strategy.

| PubMed |
| --- |
| (Dyspnea[Mesh] OR Cardiopulmonary Diseases[Mesh] OR chronic cardiopulmonary dyspnoea[Title/Abstract] OR Chronic obstructive pulmonary disease[Title/Abstract] OR heart failure[Title/Abstract] OR Asthma[Title/Abstract] OR Ischaemic heart disease[Title/Abstract] OR Interstitial lung disease[Title/Abstract] OR Pulmonary hypertension[Title/Abstract] OR pulmonary fibrosis[Title/Abstract] OR cardiomyopathy[Title/Abstract] OR valvular heart disease[Title/Abstract]) AND (Caregivers[Mesh] OR Family Caregivers[Title/Abstract] OR Informal Caregivers OR Caregiver[Title/Abstract] OR Care Givers[Title/Abstract] OR Carers[Title/Abstract] OR Family Caregiver[Title/Abstract] OR Spouse Caregiver[Title/Abstract]) |
| Embase |
| (‘Dyspnea’:ti,ab OR ‘Cardiopulmonary Diseases’:ti,ab OR ‘chronic cardiopulmonary dyspnoea’:ti,ab OR ‘Chronic obstructive pulmonary disease’:ti,ab OR ‘heart failure’:ti,ab OR ‘Asthma’:ti,ab OR ‘Ischaemic heart disease’:ti,ab OR ‘Interstitial lung disease’:ti,ab OR ‘Pulmonary hypertension’:ti,ab OR ‘pulmonary fibrosis’:ti,ab OR ‘cardiomyopathy’:ti,ab OR ‘valvular heart disease’:ti,ab) AND (‘Caregivers’:ti,ab OR ‘Family Caregivers’:ti,ab OR ‘Informal Caregivers’:ti,ab OR ‘Caregiver’:ti,ab OR ‘Care Givers’:ti,ab OR ‘Carers’:ti,ab OR ‘Family Caregiver’:ti,ab OR ‘Spouse Caregiver’:ti,ab) |
| Web of Science: |
| (TS= (Dyspnea) OR TS=(Cardiopulmonary Diseases) OR TS=(chronic cardiopulmonary dyspnoea) OR TS=(Chronic obstructive pulmonary disease) OR TS=(heart failure) OR TS=(Asthma) OR TS=(Ischaemic heart disease) OR TS=(Interstitial lung disease) OR TS=(Pulmonary hypertension) OR TS=(pulmonary fibrosis) OR TS=(cardiomyopathy) OR TS=(valvular heart disease)) AND (TS= (Caregivers) OR TS=(Family Caregivers) OR TS=(Informal Caregivers ) OR TS=(Caregiver) OR TS=(Care Givers) OR TS=(Carers) OR TS=(Family Caregiver) OR TS=(Spouse Caregiver)) |
| Cochrane Library: |
| #1 MeSH descriptor: [Dyspnea] explode all trees  #2 (Dyspnea):ti,ab,kw  #3 (Cardiopulmonary Diseases):ti,ab,kw  #4 (chronic cardiopulmonary dyspnoea):ti,ab,kw  #5 (Chronic obstructive pulmonary disease):ti,ab,kw  #6 (heart failure):ti,ab,kw  #7 (Asthma):ti,ab,kw  #8 (Ischaemic heart disease):ti,ab,kw  #9 (Interstitial lung disease):ti,ab,kw  #10 (Pulmonary hypertension):ti,ab,kw  #11 (pulmonary fibrosis):ti,ab,kw  #12 (cardiomyopathy):ti,ab,kw  #13 (valvular heart disease):ti,ab,kw  #14 MeSH descriptor: [Caregivers] explode all trees  #15 (Caregivers):ti,ab,kw  #16 (Family Caregivers):ti,ab,kw  #17 (Informal Caregivers ):ti,ab,kw  #18 (Caregiver):ti,ab,kw  #19 (Care Givers):ti,ab,kw  #20 (Carers):ti,ab,kw  #21 (Family Caregiver):ti,ab,kw  #22 (Spouse Caregiver):ti,ab,kw  #23 #1 OR #2 OR #3 OR #4 OR #5 OR #6 OR #7 OR #8 OR #9 OR #10 OR #11 OR #12 OR #13  #24 #14 OR #15 OR #16 OR #17 OR #18 OR #19 OR #20 OR #21 OR #22  #25 #23 AND #24 |
